# Role of APS reductase in biogeochemical sulfur isotope fractionation

**DOI:** 10.1038/s41467-018-07878-4

**Published:** 2019-01-09

**Authors:** Min Sub Sim, Hideaki Ogata, Wolfgang Lubitz, Jess F. Adkins, Alex L. Sessions, Victoria J. Orphan, Shawn E. McGlynn

**Affiliations:** 1 School of Earth and Environmental Sciences, Seoul National University, Seoul, 08826 South Korea; 2 Division of Geological and Planetary Sciences, California Institute of Technology, Pasadena, CA 91125 USA; 3 Max Planck Institute for Chemical Energy Conversion, Stiftstrasse 34-36, D-45470 Mülheim an der Ruhr, Germany; 4 Institute of Low Temperature Science, Hokkaido University, Sapporo, 060-0819 Japan; 5 Earth-Life Science Institute, Tokyo Institute of Technology, Ookayama, Tokyo 152-8550 Japan

## Abstract

Sulfur isotope fractionation resulting from microbial sulfate reduction (MSR) provides some of the earliest evidence of life, and secular variations in fractionation values reflect changes in biogeochemical cycles. Here we determine the sulfur isotope effect of the enzyme adenosine phosphosulfate reductase (Apr), which is present in all known organisms conducting MSR and catalyzes the first reductive step in the pathway and reinterpret the sedimentary sulfur isotope record over geological time. Small fractionations may be attributed to low sulfate concentrations and/or high respiration rates, whereas fractionations greater than that of Apr require a low chemical potential at that metabolic step. Since Archean sediments lack fractionation exceeding the Apr value of 20‰, they are indicative of sulfate reducers having had access to ample electron donors to drive their metabolisms. Large fractionations in post-Archean sediments are congruent with a decline of favorable electron donors as aerobic and other high potential metabolic competitors evolved.

## Abstract (web summary)

Changes in S-isotope ratios over time provide clues to understanding the co-evolution of Earth and its biosphere. Here the authors determine the isotope effect of the first reductive enzyme in the sulfate respiration pathway and reinterpret sedimentary S-isotope records based on this biochemical constraint.

## Introduction

Sulfate is quantitatively the most abundant water-soluble electron acceptor for biological respiration on the planet^1^, and its reduction to sulfide by microorganisms accounts for more than half of the global anaerobic degradation of organic matter^2,3^. The sulfide produced by microbial sulfate reduction (MSR) readily reacts with iron to form iron sulfide and in turn pyrite, a redox sink for sulfur and iron^4^. Thereby, this metabolism provides a linkage between the global sulfur cycle with those of carbon, oxygen, and iron. Given the presence of four stable sulfur isotopes (^32^S, ^33^S, ^34^S, and ^36^S), MSR discriminates against heavy isotopes, resulting in isotope signatures which can be recognized in modern aquatic environments and are also preserved in sedimentary sulfate and sulfide phases over geologic time^5^. Sulfur isotope fractionation has thus been used both as a diagnostic marker for sulfate reduction and as a tool to reconstruct the early evolution of sulfur metabolisms and ocean redox chemistry^6–10^. In particular, small ^34^S/^32^S fractionations between sulfate and sulfide before the great oxidation event (GOE) have been considered to reflect low-sulfate concentrations in the Archean oceans^11,12^. Such interpretations are grounded in the results of extensive culture experiments over the last half-century, which have revealed a set of relationships between microbial respiration rates, sulfate concentrations, and the kinetic and equilibrium isotope effects controlling net fractionation^13–24^. A complete mechanistic understanding of the variables that control sulfur isotope fractionation has yet to be achieved, however, in part due to a lack of information on the individual intracellular processes which cumulatively give rise to net sulfur isotope fractionation between sulfate and sulfide.

Kinetic isotope fractionation between sulfate and sulfide is, in theory, dependent on the isotope effects of the enzymes involved, the relative rates of each enzymatic reaction^25,26^, and fluxes through potential branch-points^27^. The enzymatic isotope effect is typically a reflection of the transition state formed during the reaction, and flux through a pathway and its branch-points is a complex function of physiological state and environmental conditions. To date, a few experimental constraints on metabolic sulfur fluxes have been imposed either by using the oxygen isotope exchange between sulfite and water^20–24^ or by measuring the sulfur isotope compositions of metabolic intermediates in the MSR pathway^28^. Those studies illustrated the principles underlying physiological variations in sulfur isotope fractionation, but did not give a quantitative measure of the degree of fractionation: it is the enzyme-specific isotope effects which represent the end-members of the kinetic isotope fractionation range. Knowledge of these values will allow for a quantitative interpretation of sulfur isotope fractionation arising from sulfate reduction and a linkage of isotope fractionation to the physiological state of the cell.

**Fig. 1:**
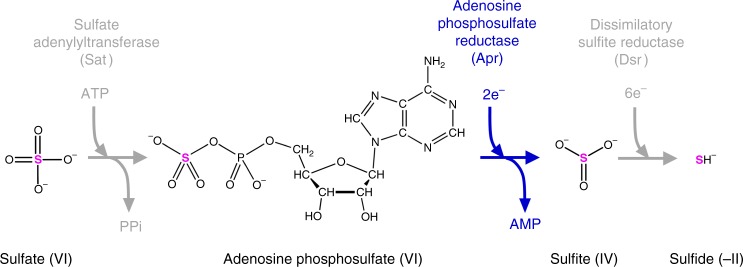
The pathway of dissimilatory sulfate reduction with the molecular structures of sulfur-containing metabolites and the enzymes catalyzing their transformation. Adenosine phosphosulfate reductase, the enzyme of interest in this study, breaks the first S–O bond in the sulfate group, which involves the transfer of two electrons

MSR to sulfide is carried out by three different enzymes: sulfate adenylyltransferase (Sat; EC 2.7.7.4), adenosine phosphosulfate (APS) reductase (Apr; EC 1.8.99.2), and dissimilatory sulfite reductase (Dsr; EC 1.8.99.5) (Fig. 1), and the assignment of kinetic isotope fractionation factors at the different steps has been based on values derived from culture experiments and equilibrium calculations^25,26^. Recently, Leavitt et al.^29^ conducted the first enzyme-specific sulfur isotope fractionation measurements, using DsrAB, a partial enzyme complex of dissimilatory sulfite reductase, and found the produced thiosulfate and trithionate to be depleted in ^34^S by 15‰ at the reduced sulfur S^0^ position of those compounds relative to the reactant sulfite. Although this reaction system lacked the DsrC subunit required to achieve full conversion of sulfite to sulfide^30^, the measured fractionation was much smaller than the previous theoretical value of ca. 50‰^26^, revealing possible discrepancies between experimental and indirect fractionation estimates and further motivating the need for the experimental assessment of enzymatic isotope effects. Remaining to be determined is the sulfur isotope fractionation by Apr, the first reductive enzyme in the MSR pathway, as well as for the full DsrABC complex. Fractionation by the Apr enzyme is predicted to exert greater control on the overall fractionation of the whole pathway as the Apr step becomes rate limiting at fast respiration rates^25,26,31^. So far, a normal isotope effect of 25‰ has been inferred for Apr^26^, but this value has never been measured.

To evaluate the contribution of Apr to microbial sulfur isotope fractionation, we determine the in vitro ^34^S/^32^S fractionation of the Apr enzyme for the first time. We incorporate the newly estimated value into a modified model of Wing and Halevy^31^, modulating the redox potential of the electron donating half-reaction and interpreting the model results in a bioenergetic context. The results enable us to propose a new sulfur isotopic constraint on the biochemistry and physiology of sulfate-reducing microbes and in doing so link growth characteristics of sulfate-reducing microbes in both modern and ancient environments to the enzymatic determinants of isotope fractionation.

## Results and discussion

### Kinetic isotope effect of the Apr enzyme

**Fig. 2:**
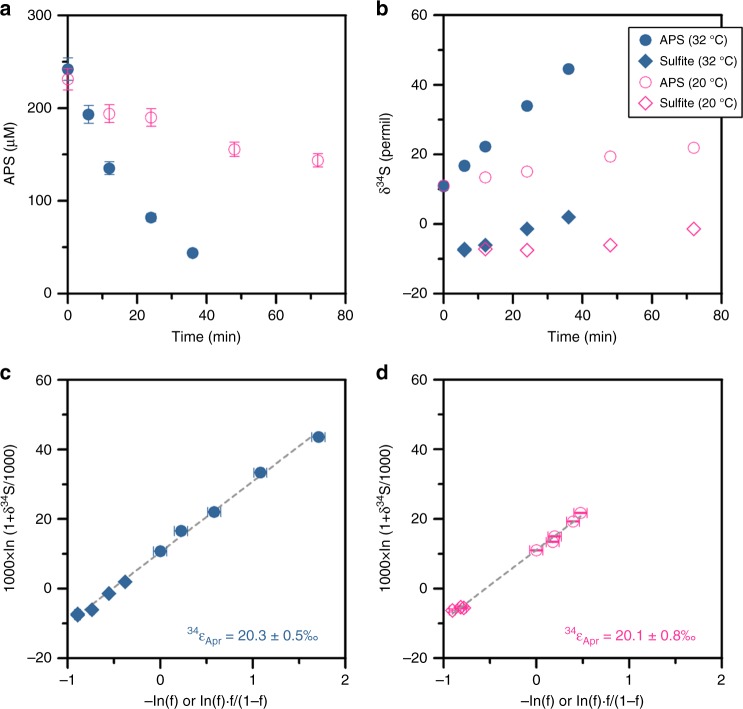
Fractionation of sulfur isotopes during the enzymatic reduction of APS at 32 and 20 °C. **a** APS consumption by APS reductase as a function of time and temperature. **b** Evolution of the *δ*^34^S values of APS and sulfite throughout the reaction at 32 °C (**c**) and 20 °C (**d**). Estimation of the kinetic sulfur isotope effect based on the Rayleigh distillation model. Values of 1000 ln(1 + *δ*^34^S(APS)/1000) and 1000 ln(1 + *δ*^34^S(Sulfite)/1000) are plotted versus −ln(*f*) and ln(*f*) × *f*/(1 − f), respectively. Dashed lines represent the linear fit, the slope of which was taken as a measure of the fractionation factor, ^34^ε_Apr_. Analytical and propagated uncertainties are shown as error bars where available

^34^S/^32^S isotope fractionation during the in vitro reduction of APS to sulfite was determined at two different temperatures, 20 and 32 °C, using purified APS reductase isolated from the mesophilic deltaproteobacteria *Desulfovibrio vulgaris* Miyazaki^32^. APS reduction was coupled to the oxidation of the electron donor methyl viologen, and the reaction at 32 °C was nearly five times faster than at 20 °C (Fig. 2a). As the reaction proceeded, remaining APS became enriched in ^34^S (Fig. 2b), consistent with a normal kinetic isotope effect. Regression analysis of *δ*^34^S values of APS and sulfite based on the Rayleigh distillation model yielded a straight line at both temperatures (Fig. 2c, d), indicating that the reactions were unidirectional without significant back reaction, and that the sulfur isotope effect remained unchanged over the course of the experiment (Supplementary Note 1, Supplementary Fig. 1). From the slope of the line, sulfur isotope effects for the reaction catalyzed by Apr (^34^ε_Apr_) were calculated to be 20.3 ± 0.5‰ and 20.1 ± 0.8‰ at 32 and 20 °C, respectively. This is the first experimental constraint on sulfur isotope fractionation by Apr, and is about 5‰ smaller than the previously inferred value of 25‰^25,26^ that was based on the equilibrium sulfur isotope fractionation between sulfate and sulfite at a physiologically relevant temperature^26^. Because the equilibrium isotope effect for reversible reactions equals the difference between kinetic isotope effects for forward and backward reactions^33^, the assumption of a 25‰ fractionation value is equivalent to assuming no kinetic fractionation in the backwards reaction. Our new measured value of 20‰ for the forward (reductive) direction suggests that Apr likely exhibits a small, inverse isotope effect when operating in the reverse (oxidative) direction. We note that an enzymatic preference toward heavy isotopes, while rare, has been previously reported in other reactions, e.g., for nitrite oxidation^34–36^.

The almost identical fractionations at two different temperatures may have implications for whole cell-derived isotope fractionation, as well as for the mechanism of enzymatic catalysis. APS reduction is thought to be a multistep reaction, which begins with nucleophilic attack on the sulfur of APS by the flavin cofactor FAD, followed by loss of AMP during collapse of the covalent APS-flavin adduct^37^. While many enzymatic reactions display weak temperature dependence of the kinetic isotope effect at the actual catalytic step, the steps in the reaction which precede or follow the catalytic step might contribute to the temperature dependence of the observed isotope effect^38–40^. The similarity of the measured sulfur isotope effects at 20 and 32 °C despite the nearly quintupled rate therefore indicates that the catalytic bond-breaking step is nearly rate-limiting for APS reduction, rather than substrate binding or product release. In contrast, fractionation of sulfur isotopes in whole cell studies has been found to change with temperature^17,41^ and also to be inversely correlated to the cell-specific sulfate reduction rate (csSRR)^13,15,18^. These differences between enzyme- and cellular-level fractionations suggest that the relationships between physiology and sulfur isotope fractionation are not rooted in the kinetics of an individual enzyme, but are instead expressed by the overall sulfate reduction pathway. We explore this next with the aid of a recently established model framework^31^ and our quantitative constraint on the sulfur isotope effect of Apr.

### Impact of Apr on sulfur isotope fractionation during MSR

**Fig. 3:**
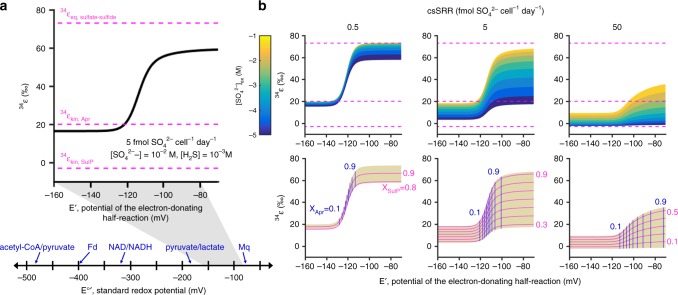
Predicted pattern of the sulfur isotope fractionation during sulfate reduction. We modify the model of Wing and Halevy^31^ to include our new experimental constraint on the sulfur isotope effect of APS reductase. Red broken lines represent the threshold values that define the allowable range of fractionation. From top to bottom, ^34^ε_eq, sulfate-sulfide_, ^34^ε _kin, Apr_, and ^34^ε_kin, SulP_ denote the equilibrium fractionation between sulfate and sulfide, the kinetic isotope effect of APS reductase, and the kinetic isotope effect of sulfate permease, respectively. **a** Sulfur isotope effect as a function of the half-reaction midpoint potential for electron donation to the terminal reductases (Apr and Dsr) under fixed sulfate concentration and cell-specific sulfate reduction rate (csSRR). Standard redox potentials for common electron donors and carriers are given for the comparison. Fd ferredoxin, Mq menaquinone. **b** Systematic exploration of the model space by varying sulfate concentrations and csSRRs. Upper panels demonstrate the influence of sulfate and intracellular redox levels on the overall fractionation at three different csSRRs. The lower panels show the reversibilities of sulfate uptake (X_SulP_) and APS reduction (X_Apr_) steps. Note that as long as the redox potential is negative enough to make the APS reduction practically irreversible, the overall fractionation cannot exceed the sulfur isotope effect imparted by APS reductase

Since MSR is a reversible multistep process, models attempting to describe the associated sulfur isotope fractionation have focused on which intermediates build up along the pathway and to what extent the enzymatic reactions interconnecting those metabolites are reversible^25,26,31^. The less reversible the reaction, the less isotope effects of the subsequent reactions will be expressed. Reversibility can be recognized as a measure of the relative degree of rate limitation at different steps in a pathway, which is ultimately set by the thermodynamic conditions of the reaction^42,43^:

1 $${\mathrm{Reversibility}}\,\left( {X} \right) = b/f = {e}^{{\Delta G}/{\mathrm{RT}}}{,}$$

where *b* and *f* denote backward and forward rates, *R* is the gas constant, *T* the temperature, and Δ*G* is the free energy change associated with the reaction. As the value of Δ*G* strongly depends on the concentrations of involved reactants and products, Wing and Halevy^31^ proposed a model with intracellular metabolite levels as the central determinant of reversibility and thus sulfur isotope fractionation. We updated their model to include the newly determined value for ^34^ε_Apr_ and used it to explore varying redox potentials of the electron donating half-reaction at constant sulfide concentration (Fig. 3). The modified model captures how both sulfate concentration and the identity of electron donors can affect the overall sulfur isotope fractionation, because the midpoint potential of the electron donor regulates the balance between reduced and oxidized forms of physiological electron carriers such as the NADH/NAD^+^ ratio^44,45^. Sulfate-reducing microorganisms can utilize a wide array of electron donors, whose potentials span the entire range of *E*’ values employed in our model^18^, and with fixed sulfate concentration and csSRR the predicted isotope fractionation increases sigmoidally as the redox potential of electron donating reaction increases (Fig. 3a). The sharp rise from one plateau to the other represents the point at which the Apr reaction changes from irreversible to reversible. Sulfate levels and csSRRs modulate the sulfur isotope fractionation primarily by altering the reversibility of sulfate uptake; as sulfate levels decrease or cells respire faster, less sulfate leaks back out of the cell, thereby drawing the isotope effect away from the large equilibrium value (Fig. 3b). Since we recently verified this reservoir effect by measuring the sulfur isotope composition of intracellular sulfate relative to ambient sulfate^28^, both modeling and experimental data suggest that sulfur isotope fractionation smaller than the 20‰ isotope effect of APS reductase can be attributed to either low-sulfate concentrations or high-respiration rates. In contrast, the modeled fractionation never exceeds the 20‰ isotope effect of APS reductase so long as the redox potential of the coupled electron transfer reaction is low enough to maintain the APS reduction step as irreversible (Fig. 3b). Sulfur isotope fractionation greater than 20‰ requires increasing the reversibility of the APS reduction step and, thus, this step exerts significant control over the expression of net isotopic fractionation by the MSR pathway^31^.

Our model predicts that the minimum sulfur isotope fractionation which can be expected when intracellular sulfate concentrations are unlimited is 20‰. This threshold established by Apr is close to the fractionation limit of ca. 17‰ measured at high metabolic rates in continuous cultures with lactate as the limiting nutrient^19^ and in marine sediments^46^. Because this minimum fractionation is also similar to the value of 15‰ obtained in the experiment with DsrAB, sulfite reduction was previously proposed as a rate-limiting step at fast respiration rates^29^. However, since sulfite reduction follows APS reduction in the metabolism, the overall sulfur isotope fractionation in the case of Dsr-imposed rate limitation should be the sum of the Dsr kinetic isotope effect and the Apr equilibrium isotope effect. Given the equilibrium sulfur isotope fractionation of 25‰ between sulfate and sulfite^26^, Leavitt et al.^29^ postulated that the formation and reduction of APS might be kinetically fast and impart a smaller sulfur isotope fractionation than commonly presumed. However, our measurement of ^34^ε_Apr_ = 20‰ negates that possibility. Thus, APS reduction—rather than sulfite reduction—appears to limit in vivo dissimilatory sulfate reduction at fast respiration rates. This is supported by the existing empirical observations. From experiments where reaction rates and isotope fractionation were measured using resting and cell extracts, Kemp and Thode^47^ concluded that the rate determining steps of sulfate reduction were prior to the final reduction of sulfite. Consistent with this rate difference, recent measurements of intracellular sulfur metabolite concentrations showed that APS—the substrate of Apr—was present at nearly ten times the concentration of sulfite within the sulfate-reducing bacterium *Desulfovibrio alaskensis* during exponential growth^28^. On the other hand, APS reduction is not always the rate-limiting step in MSR as shown by previous culture experiments with ^18^O or ^17^O-spiked water^20–22,24,48–50^. Since the half-life of direct oxygen isotope exchange between sulfate and water is about 10^9^ years at physiological temperatures^51^ and potential kinetic oxygen isotope effects are negligible compared to the amount of spike added, the primary mechanism controlling the oxygen isotope ratio of remaining sulfate is isotope exchange between sulfite and water, followed by the re-oxidation of sulfite back to sulfate^23,52,53^. In those studies, MSR was found to mediate rapid oxygen isotope exchange between sulfate and water when the accompanying sulfur isotope effect exceeded 20‰^24^ (Supplementary Fig. 2). This pattern is consistent with our model prediction that sulfur isotope fractionation greater than the Apr sulfur isotope effect of 20‰ requires a reversible, thus not necessarily rate-limiting, APS reduction step (Fig. 3).

The energy conservation and electron transfer pathways during MSR have yet to be fully elucidated, but since the reaction energies directly control reversibility and therefore the isotope effect (Eq. (1)), it may be possible to relate ^34^ε_Apr_ values to bioenergetic processes in the cell. For example, if the Apr reaction releases energy (Δ*G*_rxn_) greater than −5 kJ mol^−1^, the resulting reversibility is as small as 0.1 (Eq. (1)) and should limit isotope fractionation to 20‰ across csSRR values (Fig. 3b). Because the Δ*G* of the Apr reaction should have near-zero values to accommodate the wide range of naturally occurring fractionation, Wenk et al.^54^ recently proposed that sulfate respiration relies primarily on electron carriers with modestly negative redox potential such as menaquinone (MQH_2_/MQ, *E*°′ = −75mV). Although such a small difference between the redox potentials of electron carriers and APS (APS/HSO_3_^−^, *E*°′ = −60 mV) can contribute to maintaining reversibility in APS reduction and thereby produce larger isotope fractionation, such low energy yields make it difficult to account for the energy needed to generate a proton gradient that is presumed to be coupled to the oxidation of membrane-bound electron carriers^55,56^. However, if electron flow from physiological carriers to APS or sulfite were to be directly coupled to proton motive force generation, the reaction energies of these processes would be added to the expected energies of the APS or sulfite reduction. Because ion translocation to the outside of a chemiosmotically charged membrane is endergonic, the reversibility of a directly coupled APS or sulfite reduction step would increase as a result of the additional energy requirement (less negative Δ*G* in Eq. (1)). The small free energy change for the Apr and Dsr reactions, predicted by the sulfur isotope modeling, could then be interpreted to be a consequence of energy conservation during sulfate respiration with a resulting low net energy of the coupled reaction. Because the additional energy requirement would shift the isotope fractionation pattern visualized in Fig. 3 toward more negative redox potentials (but leave the shape of the curve unchanged), candidate electron carriers would not necessarily be limited to those with moderate redox potentials^57^. Future experiments that relate the energy state of the cell as measured by intracellular metabolite concentrations to sulfur isotope fractionation can help resolve these uncertainties and build an understanding of bioenergetic processes through isotopic analyses.

The experimental determination of Apr sulfur isotope fractionation is an important step toward a quantitative model relating the magnitude of microbial isotope fractionation to their environmental and physiological controls, but also highlights the need for further investigations into cellular processes involved in sulfate respiration. For example, the branching of APS to the PAPS assimilatory pathway^58^ is not included in the current model. It is also intriguing to consider whether the value for ^34^ε_Apr_ measured here from a single species can be applied more broadly to other sulfate-reducing microorganisms or if the isotope effects of homologous enzymes might vary significantly among different lineages.

### Constraints on the physiological state of ancient cells

**Fig. 4:**
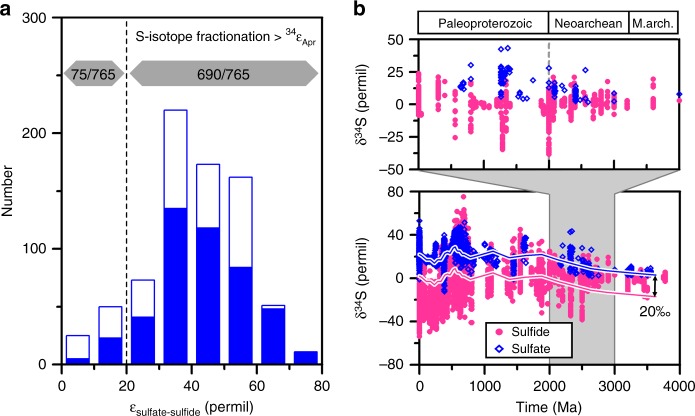
Comparison between the sulfur isotope effect of APS reductase and the sulfur isotope data compiled from previous studies of environmental and sedimentary samples. **a** Histogram showing the distribution of sulfur isotope fractionation values reported from modern terrestrial (open bars) and marine environments (filled bars) (Supplementary Data 1). Most of the measured fractionations from both environments exceed the 20‰ sulfur isotope effect of APS reductase (dashed line). **b** Compiled records of sulfate and pyrite *δ*^34^S values from sedimentary strata. The data are taken primarily from two previous compilations^5,61^ supplemented with the Archean δ^34^S data published during the last decade (Supplementary Data 2). Blue and red lines represent the seawater sulfate *δ*^34^S trend^61^ and the identical curve displaced by 20‰, respectively. The upper panel shows an expanded view of the sedimentary sulfur isotope record from the Archean to Paleoproterozoic; the dashed line in the upper panel represents 2.5 Ga. Note that the sulfur isotopic offset between sulfate and sulfide greater than 20‰ is scarce in the sedimentary rocks older than 2.5 Ga

Over the last 6 decades, nearly 100 published studies have examined sulfur isotope fractionations between sulfate and sulfide across a range of modern environments, compiling over 700 measurements in total (Fig. 4a). A wide range of sulfur isotope fractionations have been reported; however, it is clear that sulfur isotope fractionations smaller than 20‰ are quite rare in the natural environment, constituting only one tenth of the entire data set. Focusing on marine environments, the number shrinks further to only 6% of all measurements ≤20‰. Smaller sulfur isotope fractionations are more common in terrestrial environments characterized by widely variable sulfate levels, and often reflect closed-system behavior^11,59,60^. Even in these habitats, about four fifths of the data are greater than 20‰. The largest sulfur isotope fractionations occur mostly in marine environments, and this is indicative of a low-energetic driving force leading to greater reaction reversibility and isotopic fractionation as discussed above. The asymmetric distribution of environmental data around the sulfur isotope effect of Apr thus implies that electron donor availability strongly regulates MSR fractionation, whereas sulfate concentrations play an ancillary role in most modern environments.

In contrast to modern environments, sulfur isotope fractionations between sulfate and sulfide rarely exceed 20‰ in rocks older than 2.5 Ga (Fig. 4b). Such small sulfur isotope fractionations have conventionally been interpreted to represent closed-system effects of sulfate limitation^11,60^ or muted isotopic fractionation by MSR^12,62^, with low-environmental sulfate concentrations as the root cause of both. In the anoxic early oceans, however, water column sulfate reduction (open system) would likely account for a substantial fraction of pyrite formation, like those in some modern euxinic basins^63^. More importantly, neither scenario explains why the maximum sulfur isotope fractionation during the Archean would be so similar to the sulfur isotope effect of APS reductase without exceeding it.

Not only sulfate concentration but also electron donors, such as organic matter or H_2_, play a role in controlling the expression of sulfur isotope fractionation. Previous culture studies have demonstrated qualitatively that decreasing availability and reactivity of electron donors leads to larger sulfur isotope fractionation^15,18,19^, and our experimental determination of the Apr kinetic isotope effect provides quantitative support for these qualitative arguments. Based on the results above, we consider the possibility that the availability of more favorable electron donors was responsible for reduced sulfur isotope fractionation during the Archean. In an anoxic ocean harboring other microorganisms competing for reductants, using sulfate as an electron acceptor would have been a major evolutionary innovation. Sulfate-reducing microbes would likely have outcompeted ancient methanogens and acetogens for common electron donors such as H_2_, because sulfate reduction provides a greater free energy yield than CO_2_ reduction^64–66^. Considering H_2_ as an electron donor, at 100 µM concentration of sulfate and 10 nM H_2_, hydrogen oxidation coupled to sulfate reduction still yields −43.5 kJ mol^−1^, while methanogenesis provides only −13.7 kJ mol^−1^; acetogenesis is unfavorable at +20.4 kJ mol^−1^. In addition to the thermodynamic favorability of sulfate reduction when considering common electron donors, sulfate-reducing microorganisms would be able to utilize higher potential (lower energy yielding) reductants for respiration, again by virtue of delivering electrons onto sulfate, although these low-energy electron donors would likely have been utilized only if high-energy ones were substantially consumed. Competition with iron reducing microorganisms could also be considered. However, limited access to iron-bearing detrital phases yields lower rates of iron reduction in the water column^67^, and sulfate reduction often precedes iron reduction even in the low-sulfate freshwater sediments^68^. The absence of sulfur isotope fractionations greater than 20‰ is thus consistent with the hypothesis that privileged access to electron donors would be able to drive the Apr reaction irreversibly. A similar scenario could explain, at least in part, why sulfur isotope fractionation larger than 20‰ became widespread after 2.5 Ga, when the GOE, triggered by the rise of oxygenic photosynthesis, made O_2_ available as an electron acceptor^69^. With increasing oxygen concentrations, aerobic microorganisms could outcompete sulfate reducers for access to electron donors, relegating sulfate reducers to lower net energy environments. Adaptation to this thermodynamically less favorable lifestyle may have made the MSR reaction more reversible, leading to larger fractionation.

This hypothesis is yet speculative, and is not exclusive of other effects such as those from low-sulfate concentration. Indeed, simultaneous loss of electron donors and increasing sulfate concentration, both as a result of increasing atmospheric O_2_, also seems plausible in accounting for the rise in sulfur isotope fractionation values after the GOE. Regardless though, it is now clear that the electron donor is a contributing determinant to isotope fractionation during MSR, with the Apr-catalyzed reaction exerting significant control on the observed fractionation. Consequently, differences in *δ*^34^S values of sulfate and pyrite cannot be used as a quantitative constraint on sulfate concentration, e.g., in the Archean oceans^11,12^, without additional information about the reductant pool.

## Methods

### Enzymatic assay

APS reductase enzyme was purified from *Desulfovibrio vulgaris* Miyazaki as previously described^32,70^, flash frozen in 75 mM Tris-HCl buffer (pH 7.5), and stored at −80 °C for later assay. All chemicals, except for APS, were used as received from the manufacturer without further purification. The APS sodium salt was obtained from Sigma-Aldrich (CAS No. 102029-95-8), and given the relatively low-purity grade (85%) it often contained >10% sulfate (mol/mol) as impurity. The stock solution of APS (~40 mM) was prepared using degassed deionized water, split into a series of 250 μl aliquots, and stored at −80 °C until needed. Upon thawing, 50 μl of 0.2 M BaCl_2_ solution was added to each aliquot, and precipitated BaSO_4_ was removed by centrifugation. Excess Ba^2+^ was eliminated from solution by adding 100 μl of 0.1 M Na_3_PO_4_ solution, and again, the insoluble Ba_2_(PO_4_)_3_ was removed by centrifugation. After dilution, the final APS concentration in the working stock solution was set to 7.5 mM. A few tens of mM phosphate would be present in this working stock, which was not quantitatively significant because more than ten times larger amount of phosphate was added to the reaction mixture to buffer the pH (Supplementary Table 1). The in vitro APS reduction assay was carried out with methyl viologen reduced by equimolar Ti (III)-NTA as an electron donor. The reaction mixture was under anoxic and pH neutral conditions, containing 33 mM potassium phosphate, 3 mM methyl viologen, 250 µM APS, and about 10 µg ml^−1^ of enzyme in a total volume of 15 ml (Supplementary Table 1). Mixtures were incubated for 72 and 36 min at 20 and 32 °C, respectively. At every sampling point, a 2 ml subsample was extracted, preserved in a final solution of 60 mM zinc acetate^71^, flash frozen in liquid N_2_, and stored at −80 °C until the next step. Parallel control experiments without APS reductase were run simultaneously, confirming that the concentration of APS remained constant (Supplementary Table 2). Since sulfite is readily oxidized to sulfate in air in the absence of preservatives such as formaldehyde^72^, APS and sulfate, instead of sulfite^73^, were determined on a Dionex DX 500 ion chromatograph (IC) equipped with an AS11-HC column (Dionex, Sunnyvale, CA, USA), using a gradient elution with KOH as mobile phase^28^. The concentrations determined by IC are subject to an error of ±5%. For isotope analysis, the eluant fractions corresponding to sulfate and APS were collected, and APS was quantitatively converted to sulfate via hydrolysis at 65 °C for 12 h.

### Sulfur isotope analysis

The samples containing dissolved sulfate were dried on a hot plate and diluted in 5% nitric acid to a sulfate concentration of 20 µM to match the in-house working standard. Isotopic analysis was conducted on a Thermo Fisher Scientific Neptune Plus multi-collector inductively coupled plasma mass spectrometer (MC-ICP-MS), operated in medium resolution following the method previously described^74^. Samples were introduced to plasma via an ESI PFA-50 nebulizer and Cetac Aridus II desolvator. Sulfur isotope ratios of the sample and working standard were measured in 50 alternating cycles, and instrumental blank was estimated after each sample block. The mean blank value was subtracted from the measured signal for each mass. The measured ^34^S/^32^S ratio was calibrated using a linear interpolation between the two bracketing standard values. Sulfur isotope ratios are reported here using the conventional delta notation:

2 $${\mathrm{\delta }}^{34}{\mathrm{S}} = {}^{34}{\mathrm{R}}_{{\mathrm{sample}}}/{}^{34}{\mathrm{R}}_{{\mathrm{VCDT}}} - 1{,}$$

where ^34^R_sample_ and ^34^R_VCDT_ are the ^34^S/^32^S ratios of sample and Vienna-Cañon Diablo Troilite (VCDT), respectively. Our working standard was calibrated against the IAEA S-1 reference material (*δ*^34^S_VCDT_ = −0.3‰) and has a *δ*^34^S value of −1.55‰ ± 0.16 (2*σ*) on the VCDT scale. Analytic reproducibility for *δ*^34^S has been previously evaluated as ±0.2‰ (2*σ*)^74^. Measured concentrations and isotopic compositions of APS and sulfate were summarized in Supplementary Table 2. Isotopic mass balances were estimated by using a simple mixing model for a closed system:

3 $${\mathrm{\delta }}^{34}{\mathrm{S}}\left( {{\mathrm{TOT}}} \right) = \left( {{\mathrm{\delta }}^{34}{\mathrm{S}}\left( {{\mathrm{APS}}} \right) \cdot \left[ {{\mathrm{APS}}} \right] + {\mathrm{\delta }}^{34}{\mathrm{S}}\left( {{\mathrm{SO}}_4} \right) \cdot \left[ {{\mathrm{SO}}_4} \right]} \right)/\left( {\left[ {{\mathrm{APS}}} \right] + \left[ {{\mathrm{SO}}_4} \right]} \right){,}$$

where *δ*^34^S(*x*) is the sulfur isotopic composition of the total, APS, or sulfate sulfur pools, and [APS] and [SO_4_] are their molar concentrations. Calculated isotopic compositions of total sulfur varied within 10% of that of the beginning, which is a threshold value commonly used in isotope studies^75^. Closure of isotope mass balance indicates that assumption of a closed system is valid, and no unknown sulfur pool was present in our experiments. As shown in Supplementary Table 2, despite the thorough purification of APS stock solution, a trace amount of sulfate was present in the initial reaction mixture, leading to the deviation of the *δ*^34^S value of accumulated sulfate from that of sulfite produced during the assay. Given that no additional sulfate evolved in the control experiments, the contribution from an initial sulfate pool was removed via isotope mass balance:

4 $${\mathrm{\delta }}^{34}{\mathrm{S}}\left( {{\mathrm{SO}}_3} \right) = \left( {{\mathrm{\delta }}^{34}{\mathrm{S}}\left( {{\mathrm{SO}}_4} \right) \cdot \left[ {{\mathrm{SO}}_4} \right] - {\mathrm{\delta }}^{34}{\mathrm{S}}\left( {{\mathrm{SO}}_4} \right)_0 \cdot \left[ {{\mathrm{SO}}_4} \right]_0} \right)/\left( {\left[ {{\mathrm{SO}}_4} \right] - \left[ {{\mathrm{SO}}_4} \right]_0} \right){,}$$

where *δ*^34^S(SO_3_) is the isotopic composition of produced sulfite, *δ*^34^S(SO_4_) and *δ*^34^S(SO_4_)_0_ are sulfur isotope compositions of the accumulated and initial sulfate, and [SO_4_] and [SO_4_]_0_ are sulfate concentrations at the time of sampling and at time zero. Errors from isotope analysis via ICP-MS (0.2‰) and concentration measurement via IC (±5%) were propagated via first-order Taylor series expansion, resulting in an uncertainty estimate for *δ*^34^S(SO_3_) values of better than 0.5‰. Sulfur isotope fractionation factor (^34^ε_Apr_) was calculated using an approximate solution to the Rayleigh distillation equations^76^:

5 $$1000 \cdot {\mathrm{ln}}\left( {1 + {\mathrm{\delta }}^{34}{\mathrm{S}}\left( {{\mathrm{APS}}} \right)/1000} \right) = 1000 \cdot {\mathrm{ln}}\left( {1 + {\mathrm{\delta }}^{34}{\mathrm{S}}\left( {{\mathrm{APS}}} \right)_0/1000} \right) - {}^{34}\varepsilon _{{\mathrm{Apr}}} \cdot \ln f{,}$$

6 $$1000 \cdot {{\mathrm{ln}}\left( {1 + {\mathrm{\delta }}^{34}{\mathrm{S}}\left( {{\mathrm{SO}}_3} \right)/1000} \right) = 1000 \cdot {\mathrm{ln}}\left( {1 + {\mathrm{\delta }}^{34}{\mathrm{S}}\left( {{\mathrm{APS}}} \right)_0/1000} \right) + {}^{34}\varepsilon _{{\mathrm{Apr}}} \cdot \left( {f\cdot \ln f} \right)/\left( {1 - f} \right){,}}$$

where *f* is the fraction of the remaining APS, *δ*^34^S(APS)_0_ and *δ*^34^S(APS) are sulfur isotope compositions of the initial and remaining APS, respectively, and *δ*^34^S(SO_3_) is isotopic composition of produced sulfite. Using linear regression analysis, values of ^34^ε_Apr_ were obtained from the slope of -ln*f* versus *δ*^34^S(APS) and (*f*·ln*f*)/(1 − *f*) versus *δ*^34^S(SO_3_). All analytical errors were propagated via Monte Carlo simulation (*n* = 5000).

### Model for sulfur isotope fractionation during MSR

To determine the role of APS reductase in controlling the overall isotope fractionation, the estimated isotope effect of APS reductase was applied to the quantitative model for microbial sulfur isotope fractionation. While the previously published model predicted the overall fractionation as a function of the concentrations of extracellular sulfate and sulfide and the specific rate of sulfate reduction^31^, we ran the model with a constant sulfide concentration but varied redox potential for the electron carriers coupled to the MSR pathway (Supplementary Note 2, Supplementary Table 3). The sensitivity of the model results to changing sulfide levels was tested (Supplementary Figs. 3 and 4).

### Free energies of hydrogenotrophic metabolisms

Calculations were done using “eQuilibrator” at pH 7 and other reactants in those calculations were set at 100 μM^77^.

### Sulfur isotope data compilations

Sulfur isotope data from modern environments, shown as a histogram in Fig. 4, were obtained from previous compilations^19,60,78^ with addition of the recent environmental studies^79–84^. The data are binned and recalculated in Supplementary Data 1. The *δ*^34^S values of post-Archean sedimentary sulfate and sulfide are taken primarily from two previous compilations^5,61^. Recent advances in the analysis of sulfur isotope ratios, including the use of MC-ICP-MS and secondary ion mass spectrometry, have facilitated the investigation of mass-independent sulfur isotope signatures (Δ^33^S) in the Archean rocks. Since the proliferation of Archean ^33^S studies has also yielded a record of the ratio between the more abundant isotopes, ^34^S and ^32^S, we supplement the existing compilation with the Archean *δ*^34^S data collected during the last decade (Supplementary Data 2).

## Supplementary information

Supplementary Information

Description of Additional Supplementary Files

Supplementary Data 1

Supplementary Data 2

## Data Availability

Data supporting the findings of this study are available within the paper and in the supplementary information file or are available from the corresponding author upon reasonable request.
