# Supplementary material for: Role of APS reductase in biogeochemical sulfur isotope fractionation
Source: Nat Commun. 2019 Jan 9;10:44. doi: 10.1038/s41467-018-07878-4 (PMC6327049; doi:10.1038/s41467-018-07878-4)
Supplement: Supplementary file 1 — Supplementary Information [file 41467_2018_7878_MOESM1_ESM.pdf]

SUPPLEMENTARY INFORMATION

For

Role of APS reductase in biogeochemical sulfur isotope fractionation

Sim et al.

Contents

Supplementary notes including references
Four supplementary figures
Three supplementary tables

Additional Supplementary Files

File Name: Supplementary Data 1 (XLSX)
Description: Sulfur isotope fractionation in modern environments
File Name: Supplementary Data 2 (XLSX)
Description: Archean sulfur isotope data collected during the last decade

Supplementary Note 1: Unidirectional APS reduction.

Since the assay was carried out in batch reaction conditions, the irreversible nature of APS reduction under our experimental conditions must be ensured to calculate the kinetic sulfur isotope effect *via* the Rayleigh isotope fractionation model (Fig. 2). While the near linear decrease of APS with time may confirm that the reaction was unidirectional at 20℃ (zeroth-order reaction), the apparent first-order decay of APS concentration at 32℃ necessitates another means to evaluate the unidirectionality of APS reduction. Specifically, the net rate of APS consumption at 32℃ decreased from the initial reaction rate of 10.9 μM/min, with a first order rate coefficient of 0.045 min^{-1} (Supplementary Fig. 1A), which might result from either decreasing forward, increasing backward reaction rates, or both. Here we performed a simple simulation experiment for each case, showing that the present data are not compatible with the possibility that APS reduction had significant back reaction at 32℃. For simulation, a normal kinetic sulfur isotope effect of 20‰ is assigned for the reduction of APS, and the temporal evolution of the sulfur isotope compositions of APS and sulfite were calculated in accordance with the observed decay of APS concentration. When the model system assumed that the backward reaction rate is constant at zero and the decreasing forward reaction rate alone is responsible for the observed first order decay of APS concentration, the sulfur isotope compositions of APS and sulfite are aligned on a straight line with a slope of 20‰ (Supplementary Fig. 1B). In contrast, assuming that an increasing backward reaction rate contributes toward the first order decay of APS concentration, the model results deviate from the straight line as the reaction proceeds (Supplementary Figs. 1C and 1D). Such modeling results demonstrate that if there is a significant backward flux during the assay, an isotope mixing between reactant and product pools makes the measured isotope data curve away from the linear regression line on the Rayleigh plot, which is not the case in our study. A loss of enzymatic activity over time[1] is a probable cause for the observed decrease in net reaction rate, consistent with the model represented in Supplementary Fig. 1B, where no backward reaction is present.

Supplementary Note 2: Model Sensitivity Analysis

We adopt the model of Wing and Halevy[2] to further elucidate the role of APS reductase in determining the sulfur isotope fractionation at a cellular level. For details of the equations and parameters used in this model, please see the original paper[2] and Supplementary Table 3.

In their model, sulfate uptake and APS reduction are the most important determinants of isotope fractionation, since these two reactions vary considerably from reversible to nearly unidirectional under a range of physiological conditions. In contrast, the formation of APS and sulfite reduction steps are nearly reversible across almost all cellular states, and so contribute less to varying sulfur isotope fractionation. The relatively irreversible nature of the Apr step under many conditions can be partly explained by the fact that the standard reduction potential of APS to sulfite is ~60mV more positive than that of sulfite to sulfide at neutral pH. Any model that uses a common electron donor for both reactions will necessarily have APS reduction as being less reversible. Experimental determination of the sulfur isotope effect of Apr – the first reductive and S-O bond breaking enzyme in the MSR pathway - therefore immediately places an improved constraint on a major parameter of the model.

Wing and Halevy[2] modeled sulfide concentrations ranging from 10^{-5} to 10^{-2} M, but we hold it as a constant at 10^{-3} M. Instead, the redox midpoint potential of the half reaction that donates electrons to the terminal reductases is added as a free variable. Our model thus has three tunable parameters: cell-specific sulfate reduction rate (csSRR), extracellular sulfate concentration, and redox potential of electron carriers. In the previous calculations[2], menaquinone served as the electron donor for both APS and sulfite reductases, and since its midpoint potential ($E^{o\prime}$= -74 mV) is not negative enough to induce sulfite reduction ($E^{o\prime}$= -116 mV) at standard state, the ratio of reduced to oxidized menaquinone is set to be 100:1, generating a more favorable redox potential (E'= -129 mV). With the ratio being halved or doubled, their sensitivity tests briefly show that the model fractionation is sensitive to the free energy change associated with the electron-donating reaction[2]. To exemplify the effect of electron donors on the $^{34}\varepsilon$ patterns, we do not change the default ratio of 100 but instead increase the reduction potential of electron-donating half reaction from -160 to -90 mV (Fig. 3). As a result, there are three threshold values that shape the pattern of sulfur isotope fractionation, two of which are set by the kinetic isotope effect of sulfate permease and by the equilibrium fractionation between sulfate and sulfide. The former indicates the minimum sulfur isotope fractionation when sulfate uptake is limiting and irreversible[3-5], and can be visualized in Fig. 3B where sulfur isotope fractionation is depressed at high csSRR. The latter suggests the maximum is associated with the thermodynamic lower limit of microbial metabolism[6]. In between these two scenarios is a range of physiological conditions where the modeled fractionation never exceeds the 20‰ isotope effect of APS reductase so long as the redox potential of the coupled electron transfer reaction is low enough to maintain the APS reduction step as irreversible.

Because the model requires that a combination of tunable parameters yields the concentrations of all metabolites greater than 1 nM[2], we further monitor the intracellular concentrations of sulfate, APS, sulfite, and pyrophosphate (PPi) during the model runs. Although the concentrations of all three sulfur metabolites are maintained greater than 1 nM, calculated PPi concentrations become sub-nanomolar as the overall reaction approaches equilibrium. Regions where calculated PPi concentrations are physiologically unlikely, however, do not change the results of our model qualitatively (Supplementary Fig. 3). Since the pattern of sulfur isotope fractionation in the main text (Fig. 3) assumes a sulfide concentration of 10^{-3} M, we also examine the sensitivity of our results to the chosen sulfide concentration. With the sulfide concentration increasing or decreasing 20-fold, the predicted pattern of sulfur isotope fractionation does vary modestly but not in a way that alters the three bracketing values (Supplementary Fig. 4).

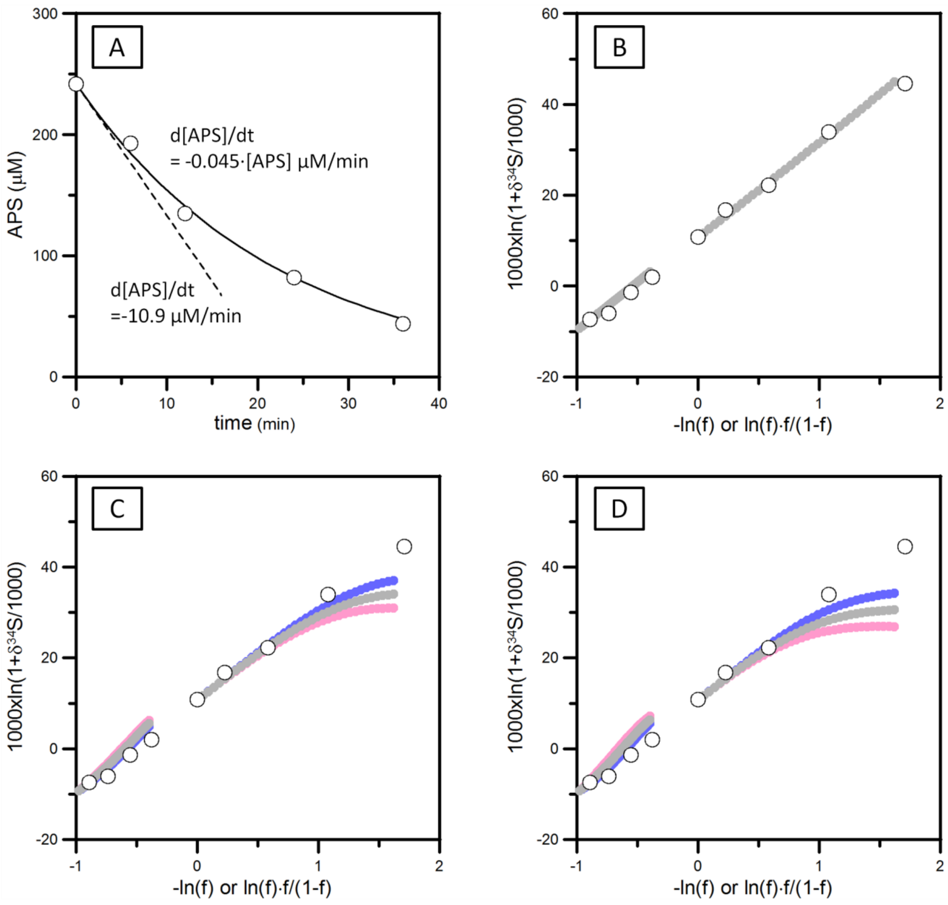

Supplementary Fig. 1. Rayleigh isotope distillation model and unidirectionality of APS reduction during the assay. (A) Measured (empty circle) and modeled APS (solid line) concentrations over time at 32°C. The net rate of APS consumption appears to decrease from the initial reaction rate of 10.9 µM/min with the first order rate coefficient of 0.045 min^{-1}. (B) Measured (empty circle; Fig. 2C) and modeled isotope trends (small gray circle) on the Rayleigh plot. This model system assumes that the backward reaction rate is constant at zero and the decreasing forward reaction rate alone is responsible for the observed first order decay of APS concentration. For model calculation, a normal kinetic sulfur isotope effect of 20‰ is assigned for the forward reaction. (C) Model results with the assumption that decreasing forward and increasing backward fluxes equally contribute toward the first order decay of APS concentration. A normal isotope effect of 20‰ is assigned for the forward reaction, but three different isotope effects (-5, 0, and 5‰; blue circles, gray circles, and red circles) are used for the backward reaction to test the model sensitivity. (D) Model results derived from an assumption of first order decay of APS concentration resulting from an increasing backward reaction rate, while the rate of forward reaction is constant at 10.9 µM/min.

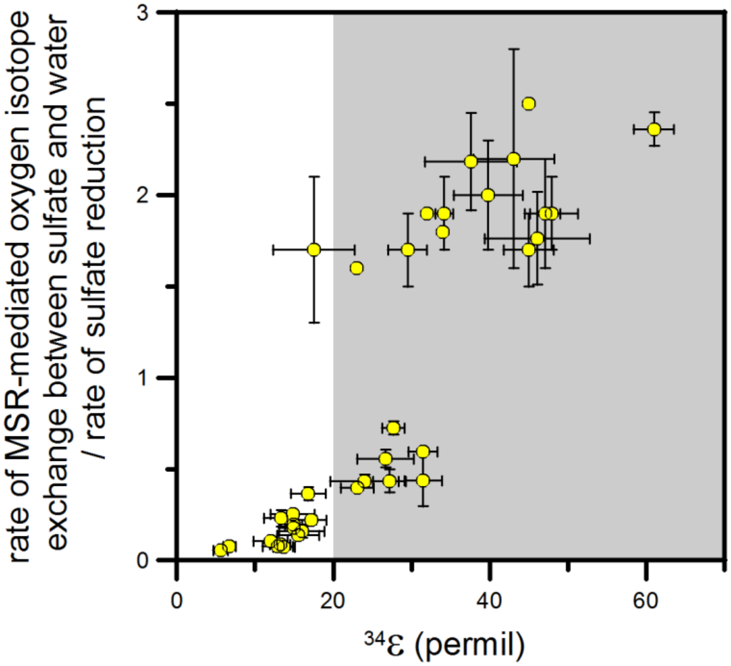

Supplementary Fig. 2. Relationship between the sulfur isotope fractionation and the ratio of rate of oxygen isotope exchange between sulfate and water to rate of sulfate reduction (modified after ref[7]). Rapid oxygen isotope exchange (y-axis values greater than 1) occurs predominantly with accompanying sulfur isotope fractionation greater than 20‰, while smaller sulfur isotope fractionations severely limit the extent of oxygen isotope exchange between sulfate and water. Error bars refer to the 95% confidence interval.

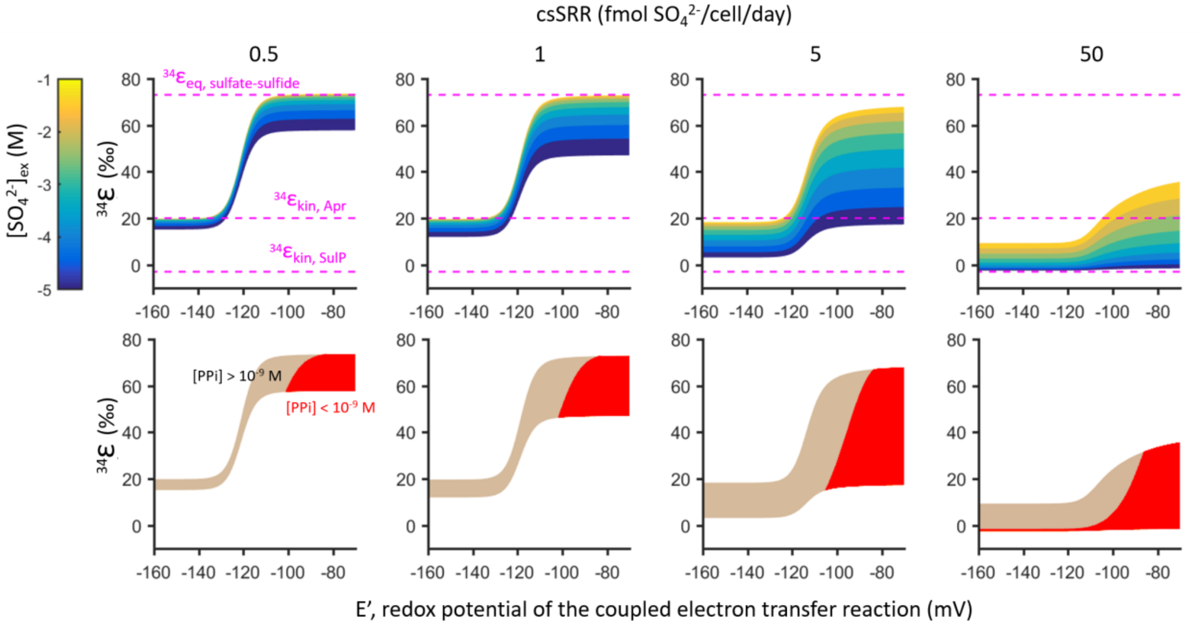

Supplementary Fig. 3 Predicted pattern of the sulfur isotope fractionation shown in Figure 3 in the main text. The regions where calculated PPi concentrations are physiologically unlikely (< 1 nM) are shown as red-colored fields

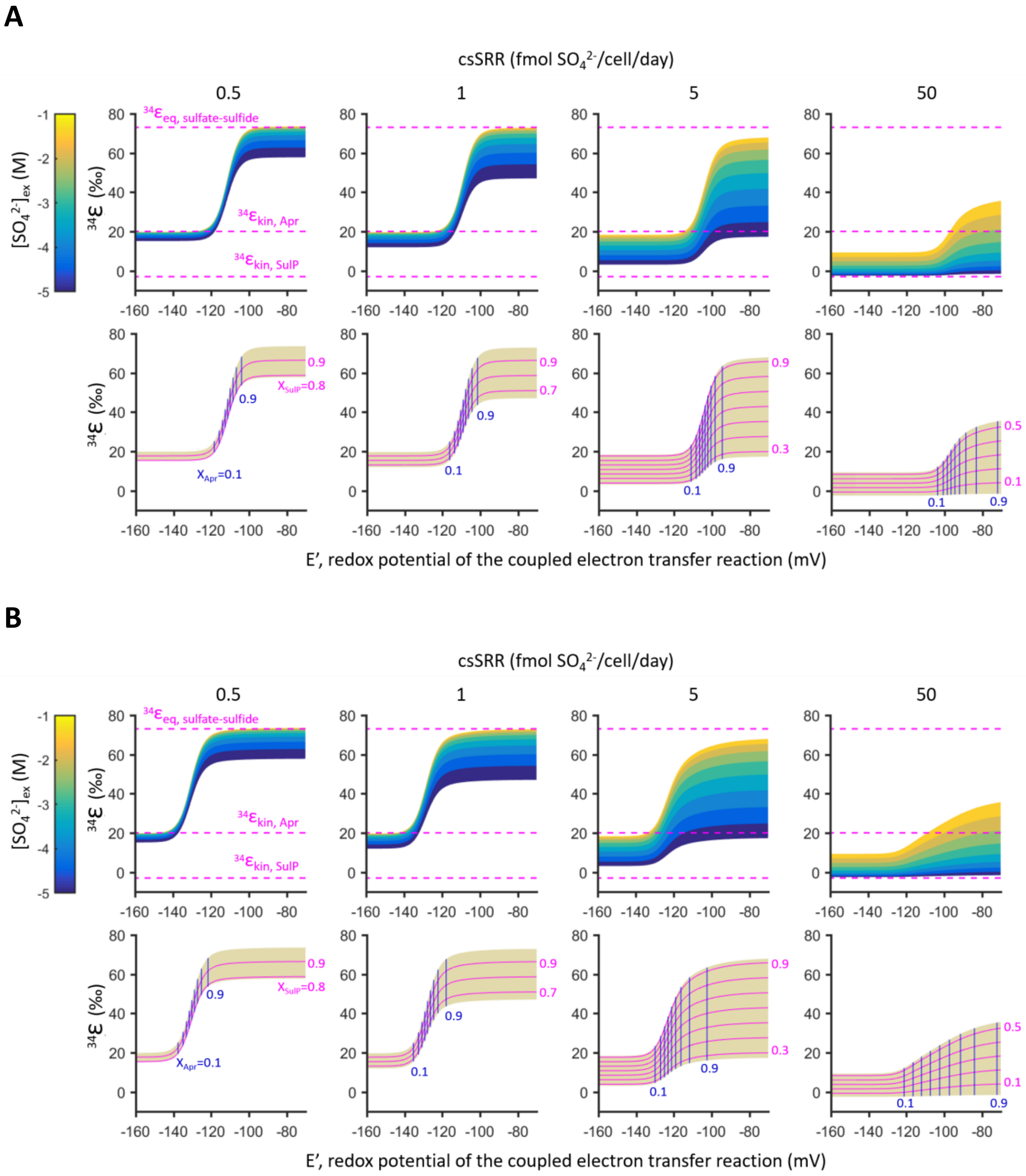

Supplementary Fig. 4 Sensitivity of model results to sulfide concentration. Predicted patterns of sulfur isotope fractionation are shown for two different sulfide concentrations of 50 μM (panel A) and 20 mM (B).

Assay mixture

Components	*Concentration (mM)*
2.5 ml 0.2 M potassium phosphate pH 7	33.3
3 ml 15 mM methyl viologen reduced with Titanium(III)-NTA	3
0.5 ml 7.5 mM APS	0.25
9 ml Nanopure water	

Procedure

15 ml assay mixture
20 µl APS reductase stock (9 µg/µl)
100% N_2 atmosphere
Temperature, 32℃ or 20°C

Supplementary Table 1. Assay conditions for APS reductase

T (°C)	time (min)	concentration (uM) APS			concentration (uM) sulfate + sulfite			δ34S (permil) APS			δ34S (permil) sulfate + sulfite			δ34S (permil) sulfite			δ34S (permil) total sulfur
32	0	242.0	±	12.1	14.2	±	0.7	10.8	±	0.2	9.30	±	0.2				10.8
	6	193.2	±	9.7	60.9	±	2.9	16.7	±	0.2	-3.36	±	0.2	-7.2	±	0.4	11.9
								16.8	±	0.2	-3.62	±	0.2	-7.5	±	0.5	11.9
	12	135.2	±	6.8	104.6	±	5.2	22.3	±	0.2	-3.97	±	0.2	-6.0	±	0.3	10.8
	24	82.1	±	4.1	167.2	±	8.4	33.9	±	0.2	-0.47	±	0.2	-1.4	±	0.2	10.9
	36	43.8	±	2.2	213.3	±	10.7	44.6	±	0.2	2.48	±	0.2	2.0	±	0.2	9.7
	38 (control)	247.9	±	12.4	15.1	±	0.8	10.9	±	0.2							
20	0	231.2	±	11.6	11.4	±	0.6	11.1	±	0.2	7.20	±	0.2				10.9
	12	194.0	±	9.7				13.4	±	0.2							
	24	189.8	±	9.5	41.4	±	2.1	15.1	±	0.2	-2.53	±	0.2	-6.3	±	0.5	11.9
	48	155.6	±	7.8	82.3	±	4.1	19.4	±	0.2	-3.63	±	0.2	-5.4	±	0.3	11.5
	72	143.7	±	7.2	101.4	±	5.1	22.0	±	0.2	-4.10	±	0.2	-5.5	±	0.3	11.2
	75 (control)	233.5	±	11.7	13.2	±	0.7	10.7	±	0.2	8.20	±	0.2				10.5

Supplementary Table 2. Concentration and sulfur isotope data from the enzymatic assay

Step	Equilibrium isotope fractionation ($^{34}\alpha^{eq}$)	Kinetic isotope fractionation ($^{34}\alpha^{kin}$)
SulP (sulfate, out - sulfate, in)	1.000	0.997
Sat (sulfate - APS)	1.000	1.000
**Apr (APS - sulfite)**	1.025	**1.020**
Dsr (sulfite - sulfide)	1.048	1.053

Supplementary Table 3. Equilibrium and kinetic fractionation factors used in the model. The isotope fractionation factor between two pools A and B is $^{34}\alpha_{A\text{-}B} = (^{34}S/^{32}S)_A/(^{34}S/^{32}S)_B$. The Apr kinetic fractionation was determined in this study (bold), and all other values were taken from ref[8].

Supplementary references

1. Suter, M. et al. Adenosine 5′-phosphosulfate sulfotransferase and adenosine 5′-phosphosulfate reductase are identical enzymes. *J. Biol. Chem.* **275**, 930-936 (2000).
2. Wing, B. A. & Halevy, I. Intracellular metabolite levels shape sulfur isotope fractionation during microbial sulfate respiration. *Proc. Natl. Acad. Sci.* **111**, 18116-18125 (2014).
3. Sim, M. S., Paris, G., Adkins, J. F., Orphan, V. J. & Sessions, A. L. Quantification and isotopic analysis of intracellular sulfur metabolites in the dissimilatory sulfate reduction pathway. *Geochim. Cosmochim. Acta* **206**, 57-72 (2017).
4. Harrison, A. G. & Thode, H. G. Mechanism of the bacterial reduction of sulphate from isotope fractionation studies. *Trans. Faraday Soc.* **54**, 84-92 (1958).
5. Habicht, K. S., Gade, M., Thamdrup, B., Berg, P. & Canfield, D. E. Calibration of sulfate levels in the Archean ocean. *Science* **298**, 2372-2374 (2002).
6. Sim, M. S., Bosak, T. & Ono, S. Large sulfur isotope fractionation does not require disproportionation. *Science* **333**, 74-77 (2011).
7. Brunner, B. et al. The reversibility of dissimilatory sulphate reduction and the cell-internal multi-step reduction of sulphite to sulphide: insights from the oxygen isotope composition of sulphate. *Isotopes Environ. Health Stud*. **48**, 33-54 (2012).
8. Brunner, B. & Bernasconi, S. M. A revised isotope fractionation model for dissimilatory sulfate reduction in sulfate reducing bacteria. *Geochim. Cosmochim. Acta* **69**, 4759-4771 (2005).
