# Supplementary material for: Role of APS reductase in biogeochemical sulfur isotope fractionation
Source: Nat Commun. 2019 Jan 9;10:44. doi: 10.1038/s41467-018-07878-4 (PMC6327049; doi:10.1038/s41467-018-07878-4)
Supplement: Supplementary file 2 — Description of Additional Supplementary Files [file 41467_2018_7878_MOESM2_ESM.docx]

**Description of Supplementary Information Files**

**File Name:** Supplementary Data 1

**Description:** Sulfur isotope fractionation in modern environments.

**File Name:** Supplementary Data 2

**Description:** Archean sulfur isotope data collected during the last decade.
